# Editorial: A global perspective on suicidal behaviour and ideation: demographics, biomarkers and treatment

**DOI:** 10.3389/fpsyt.2023.1218831

**Published:** 2023-05-24

**Authors:** Qi Wang

**Affiliations:** School of Graduate Studies, Lingnan University, Hong Kong, Hong Kong SAR, China

**Keywords:** suicide, demographics, treatment, assessment, review, evidence-based practice

Suicide is an alarming public health issue that transcends geographical, cultural, and socioeconomic boundaries (1). The complexity of its causes and manifestations necessitates the continuous exploration and understanding of its various aspects. In recent decades, suicide has become a leading cause of death worldwide, with devastating social, psychological, and economic consequences (2). Despite the increasing recognition of this public health crisis, there is a persistent need for a comprehensive understanding of suicidal behavior and ideation in order to develop more effective prevention and intervention strategies. In this editorial, we will explore the complex and multifaceted nature of suicide, examining its demographics, biomarkers, and treatment from a global perspective. By shedding light on this challenging subject, we aim to contribute to the ongoing conversation surrounding mental health and stimulate further research and collaboration across disciplines and borders. The focus of this Research Topic is understanding suicidal behavior and ideation from a global perspective. In this special issue, we have gathered eight articles that contribute to this ongoing dialogue, providing valuable insights into suicide risk factors, assessment, and prevention strategies across diverse populations and contexts. The collection of papers includes brief research reports, original research, perspectives, reviews, and retrospective case series. The contributions come from a variety of disciplines and are based on diverse research populations in Bulgaria, Australia, Spain, Hungary, and China.

Stoychev et al. brief report sheds light on the vulnerability of psychiatric patients. This study examines a decade's worth of data from a Bulgarian registry and concludes that mood disorders, schizophrenia, anxiety disorders, substance use disorders, and organic conditions are the most prevalent diagnoses associated with suicide. The authors also demonstrate that male gender, single or divorced marital status, early onset of illness, co-occurring substance abuse, and inferior educational attainment (for patients below the age of 70) were significantly associated with an earlier age of suicide. In contrast, there was no significant association between past suicide attempts and psychiatric hospitalizations, comorbid somatic conditions, or unemployment. Another important finding from this study was that a substantial proportion of patients contacted psychiatric services in the year prior to their suicide, with nearly half of these contacts occurring within 30 days of the accident. This study identifies significant suicide risk factors, including sociodemographic and illness-related factors. These findings can be utilized to more effectively develop targeted interventions and allocate resources to cater to the needs of high-risk populations.

Another important risk factor, psychological pain, was identified and summarized in Baryshnikov and Isometsa review of this concept. This article explores the relationship between psychological pain and suicidal behavior. By outlining the history of the concept, the definition of psychological pain, and the tools developed for its measurement, this review summarized the empirical research on psychological pain in relation to suicidal behavior and suggested future directions for clinical research on psychological pain and suicidal behavior. Thus, mental health professionals can develop targeted interventions that address this underlying issue after fully understanding the role of psychological pain in the development and maintenance of suicidal thoughts and actions.

Besides the studies conducted in Western countries, the retrospective case series analysis of 1,091 Chinese inpatients with major depressive disorder (MDD) by Liu et al. identified risk factors for suicide behaviors among MDD patients, including female gender, history of major mental trauma, impulsivity, family history of suicide, and severity of depression. By examining the nuances of these factors, mental health professionals can develop targeted interventions to better support patients with MDD who are struggling with suicidal thoughts and behaviors.

Meanwhile, two other articles identified two protective factors for suicide prevention. Folgado et al. study investigates the dynamic nature of suicidal thoughts among Spanish college students using the method of ecological momentary assessment. A total of 737 participants joined the study and were followed for 6 months, and suicide ideation was assessed 14 times during this period. This innovative approach allows researchers to track fluctuations in suicidal ideation in real-time, offering valuable insights into the factors that contribute to the onset and persistence of these thoughts. This study found that one latent dimension of risk factors (higher levels of thwarted belongingness, perceived burdensomeness, depressive symptoms, negative affect, and emotional suppression) best represented the group with moderate levels of suicidal ideation, whereas another latent dimension of protective variables (positive affect, cognitive reappraisal, and life purpose) best represented the group with lower levels of suicidal ideation. This study indicates that students with a positive outlook on life and the capacity to reevaluate negative beliefs are less likely to have suicidal thoughts. Therefore, having a sense of purpose in life would be a protective factor against suicidal ideation. This understanding can lead to the development of adaptive interventions that address the needs of college students at risk.

The original study conducted by Katzenmajer-Pump et al. highlights the unique challenges faced by adolescents with ADHD in Hungary. In total, 89 adolescents with ADHD and 96 without ADHD were enrolled in this study. By identifying feelings of worthlessness as a significant risk factor for suicide in this population, this study draws the attention of clinicians to the importance of recognizing “worthlessness” for suicide prevention in adolescents with ADHD. In addition, the results support previous research indicating that depression and anxiety symptoms mediate the relationship between ADHD and suicidal ideation and planning. These findings emphasize the significance of ADHD co-morbidities with depression and GAD, as well as their impact on suicidal ideation and planning. The study also underscores the need for tailored prevention strategies that address the emotional and psychological challenges faced by adolescents with ADHD.

Facing the risk and protective factors of suicide behavior, the original study conducted by Hawgood et al. in Australia focuses on the importance of equipping mental health professionals with the tools and knowledge to effectively assess and respond to suicidality. The study demonstrates the positive impact of the Systematic Tailored Assessment for Responding to Suicidality (STARS) protocol training, emphasizing the need for ongoing education and training in suicide prevention and intervention. The STARS participants who reported greater perceived capability at baseline were the professionals who had significantly greater formal and informal training, more years of experience in suicide prevention, were more likely to have witnessed a client suicide or suicide attempt, and reported fewer suicide risk assessment-related fears. From pre- to post-training, this study discovered significant positive effects of STARS training on clinician competencies (attitudes, perceived capability, and declarative knowledge). Perceived capability and declarative knowledge underwent the most notable changes following STARS training, which demonstrates the effectiveness of STARS training on improving attitudes, perceived capability, and declarative knowledge in mental health professionals. This study offered an evidence-based training model for mental health professionals for suicide prevention.

The perspective study conducted by Rudd and Bryan also explores how recent research advancements on suicide prevention can be incorporated into clinical practice. This study identifies the research and practice gap that clinicians frequently grapple with and how to incorporate recent advances into practice in an efficient and effective manner in daily practice. Then, five critical domains were proposed to facilitate clinicians' risk formulation processes. First of all, the author suggested that screening and standard assessment methods are useful but restricted parts of a suicide risk assessment procedure due to their inaccuracy, unreliability, and lack of adequacy in capturing the natural temporal dynamics of suicidal thinking. Therefore, healthcare decisions should use many data points, such as behavioral observations, to better assess patient risk and understand individuals' shifts and cycles of suicide risk. Different clinical interview questions, especially focused interview questions based on recent research, may help improve risk formulation and clinical decision-making. Moreover, other constructs other than suicidal ideation, such as perceived burdensomeness, acquired capability, and identity-based hopelessness, should also be taken into consideration. During counseling, treatment hesitancy and non-adherence with clinical care recommendations shall be dealt with, as well as assessing the wish to live and the wish to die. This study recognized and addressed the importance of observed clinical discrepancies in the risk formulation process.

Finally, the perspective study conducted by Ochuku et al. discusses the importance of decriminalizing suicide in low- and middle-income countries. To reduce suicide rates, timely implementation of effective policies and legislation must accompany research findings. As an outmoded practice that may do more harm than good, it is essential to repeal laws that criminalize suicide attempts in countries where they remain illegal. Methods of suicide prevention supported by empirical evidence include means restriction, mental health literacy improvement, access to psychosocial support, and responsible media coverage. It is anticipated that these efforts will assist individuals contending with suicidal ideation on a national and global scale.

## Conclusion

In conclusion, suicidal behavior and ideation are global public health issues that deserve more attention and research. There are opportunities to gain valuable insights by examining trends across countries and cultures. Improved treatment and prevention strategies that are tailored to specific demographics and cultures are also urgently needed. A coordinated global effort across researchers, healthcare systems, and policymakers will be required to make progress on reducing the rates of suicide worldwide. This special issue offers a comprehensive exploration of the intricacies of suicide, emphasizing the need for ongoing research, tailored interventions, and policy changes from a global perspective. Demographic factors, such as age, gender, and cultural background, play an important role in shaping individuals' experiences of suicidal thoughts and behaviors. Finally, effective treatment of suicidal behavior and ideation requires a holistic approach that addresses the underlying psychological, social, and environmental factors that contribute to the problem. By deepening our understanding of the various facets of suicide and its prevention, we can work collaboratively to address this significant public health issue and provide life-saving support to those most vulnerable. Moreover, by taking a comprehensive and collaborative approach, we can work toward reducing the incidence of suicidal behavior and ideation and improving the wellbeing of individuals and communities worldwide. Overall, a global perspective incorporating epidemiological, biological, and therapeutic approaches is a key to advancing our understanding and management of suicidal behavior.

Suicide is a preventable tragedy. We thank the research teams highlighted in this special topic for their contributions and encourage them to continue generating knowledge that will significantly improve our understanding of suicide, its causes, and its prevention. We trust that the Journal's readers will find this special topic timely and interesting, and that the results presented here will aid in decision-making on multiple levels.

## Author contributions

The author confirms being the sole contributor of this work and has approved it for publication.
